# Antagonist Targeting microRNA-155 Protects against Lithium-Pilocarpine-Induced Status Epilepticus in C57BL/6 Mice by Activating Brain-Derived Neurotrophic Factor

**DOI:** 10.3389/fphar.2016.00129

**Published:** 2016-05-31

**Authors:** Zhengxu Cai, Song Li, Sheng Li, Fan Song, Zhen Zhang, Guanhua Qi, Tianbai Li, Juanjuan Qiu, Jiajia Wan, Hua Sui, Huishu Guo

**Affiliations:** ^1^Department of Neurology, The First Affiliated Hospital of Dalian Medical UniversityDalian, China; ^2^Center for Translational Research on Neurological Diseases, The First Affiliated Hospital of Dalian Medical UniversityDalian, China; ^3^Central Laboratory, The First Affiliated Hospital of Dalian Medical UniversityDalian, China; ^4^Institute of Basic Research of Integrative Medicine, Dalian Medical UniversityDalian, China

**Keywords:** antagonist, brain-derived neurotrophic factor, epilepsy, microRNA-155, lithium-pilocarpine

## Abstract

Epilepsy is a severe brain disorder affecting numerous patients. Recently, it is inferred that modulation of microRNA-155 (miR-155) could serve as a promising treatment of mesial temporal lobe epilepsy. In the current study, the therapeutic potential of miR-155 antagonist against temporal lobe epilepsy (TLE) was evaluated and the underlying mechanism involved in this regulation was explored. TLE model was induced by lithium-pilocarpine method. The effect of miR-155 antagonist on epilepticus symptoms of TLE mice was assessed using Racine classification and electroencephalogram (EEG) recordings. The expression of brain-derived neurotrophic factor (BDNF) and its association with miR-155 were also assessed with a series of experiments. Our results showed that level of miR-155 was significantly up-regulated after induction of TLE model. Based on the results of EEG and behavior analyses, seizures in mice were alleviated by miR-155 antagonist. Moreover, administration of miR-155 antagonist also significantly increased the level of BDNF. The results of dual luciferase assay and Western blotting showed that miR-155 antagonist exerted its action on status epilepticus by directly regulating the activity of BDNF. Taken all the information together, our results demonstrated that miR-155 antagonist might firstly induce the expression of BDNF, which then contributed to the alleviation of epilepsy in the current study.

## Introduction

Epilepsy is a serious chronic and debilitating brain disorder characterized by paroxysmal bursts of activity within cortical neurons (Matsumoto and Marsan, 1964). Although epilepsy frequently occurs in childhood, it can affect people of whole age (Huang et al., 2015). Patients with epilepsy are generally impaired by intermittent infuriation in movement, feeling, or consciousness which is caused by the suddenly synchronous and excessive discharges in cortical neurons (Brodie and French, 2000). Currently, resective surgeries for focal epilepsy and new-generation antiepileptic drugs (AEDs) are the major treatments for patients with epilepsy (Kawai, 2015). However, both methods have some following side effects such as headache, dizziness, fatigue, and ataxia (Brodie and Dichter, 1996). Additionally, more than 20% of epilepsy patients continue to suffer from seizures even after being subjected to above mentioned therapies. Thus, identification of some novel biological markers for development of therapeutic strategies against epilepsy is demanding prompt solution.

With the development of genetic discoveries and genetic techniques, molecular causes of epilepsy has been comprehensively revealed during the past several years (Buiting et al., 1995; Buiting, 2010; Lee and Heo, 2014; El Achkar et al., 2015). Emerging evidence infers the participation of microRNAs (miRs) in the lesion of types of epilepsy (Li et al., 2014). MiRs belong to an endogenous class of small non-coding RNAs and take their actions through inhibition of translation levels of specific genes or fracture of targeted mRNAs (Schirle and MacRae, 2012). Members of miRs play crucial roles in the regulation of gene expressions and functional gene networks (Schratt, 2009). The involvement of miRs in neurological diseases has been highlighted: in response to brain injuries such as acute neurological disorders and prolonged seizures, the levels of several miRs alter chronically (Aronica et al., 2010; Liu et al., 2010; Hu et al., 2011). Therefore, potency of miRs as clinical biomarkers for detection of neurological diseases is extensively investigated (de Planell-Saguer and Rodicio, 2011). For studies regarding roles of miRs in epilepsy, understating the changing pattern and therapeutic potential of these indicators has become central subjects. In recent study of Jimenez-Mateos et al. (2015), the authors have demonstrated the vital function of miR-134 in alleviating pilocarpine-induced status epilepticus: the research provides compelling evidence that application of antagonist against miRs significantly suppresses seizures in an animal model. Furthermore, another multifunctional miR, miR-155, is proved to possess the capability to mediate acute inflammatory response associated with activation of Toll-like receptors that is a suspected feature of epilepsy onset (Tili et al., 2007; Maroso et al., 2011). The role of miR-155 in mesial temporal lobe epilepsy (MTLE) is also verified in the study of Ashhab et al. (2013), which infers that modulation of miR-155 may be an alternative therapy for treatment of MTLE. Moreover, miR155 is capable of modulating the activity of brain-derived neurotrophic factor (BDNF) that is critical to the normal development of the CNS and contributes to the development of temporal lobe epilepsy (TLE; Scharfman, 2005; Varendi et al., 2014). Such interaction between miR-155 and BDNF may represent a potential mechanism through which miR-155 functions on the onset of epileptic disorders. However, unlike studies on miR-134, the potential of miR-155 as a novel therapeutic target for epileptic disorders is only partially revealed. Considering the determinant role of miR-155 in numerous biological processes, it is reasonable to explicitly explore the function of miR-155 in epilepsy and to assess its regulation as an anti-epilepsy strategy.

In the present study, TLE model was induced by lithium-pilocarpine method. Thereafter, expressions of miR-155 at mRNA and protein levels were detected with quantitative real-time PCR (qPCR) and Western blotting assay. The effect of miR-155 antagonist on epilepsy mice was evaluated by behavior analyses and electroencephalogram (EEG) recording. The expression of BDNF and its connection with miR-155 production in epilepsy were also determined. Findings in the present study could assess the potential of miR-155 modulation as a novel anti-epilepsy therapy and provide a preliminary explanation on the mechanism driving the alleviative effect of miR-155 antagonist against epileptic disorders.

## Materials and Methods

### Chemicals and Animals

Lithium, pilocarpine, scopolamine, and diazepam were purchased from Sigma Chemical, Co. (Calt. No. L4408-100G, P6503-5G, S8502-1G, D0899, St. Louis, MO, USA). MiR-155 antagonizing sequence and a non-targeting scrambled version of the antagonist were purchased from GenePharma, Co. Ltd., (Shanghai, China). Antibodies against BDNF and β-actin were purchased from Santa Cruz (Calt. No. sc-546 and No. sc-47778, Shanghai, China). 8-weeks-old male C57BL/6 mice (weighing from 18 to 25 g) were provided by Experimental Animal Center of China Medical University and housed in cages at 20–25°C with a constant humidity (55 ± 5%) with water and food available *ad libitum*. All animal experiments were conducted in the accordance with the Institutional Animal Ethics Committee and Animal Care Guidelines for the Care and Use of Laboratory Animals of The First Affiliated Hospital of Dalian Medical University.

### Animal Grouping and Induction of TLE Models

Forty-eight mice were employed and randomly divided into four groups (12 for each group): (A) Control group, healthy C57BL/6 mice. (B) TLE group, mice underwent surgical processes to induce TLE. (C) CK group, TLE mice pre-treated with 0.12 nmol non-targeting antagonist (2 μL). (D) Antagonist group, TLE mice pre-treated with 0.12 nmol miR-155 antagonist (2 μL). For TLE induction, mice were anesthetized with pentobarbital sodium (50 mg/kg) and placed in stereotaxic frame. After a midline incision, a guide cannula was affixed to the surface of the brain for the intracerebroventricular injection (coordinates from Bregma were AP, -0.3 mm; L = -1.0 mm; and V = -2.0 mm). Mice in CK and antagonist groups were received injection of specific agents while mice in the other two groups received artificial cerebrospinal fluid (2 μL) instead. 24 h later, mice in TLE, CK, and antagonist groups were administrated with 3 mEq/kg lithium for 18 h and then subcutaneously injected with 1 mg/kg scopolamine. After additional 30 min, 340 mg/kg pilocarpine was intraperitoneally injected into mice in TLE, CK, and antagonist groups to induce epilepsy. 90 min after seizure attack, mice in TLE, CK, and antagonist groups were given 6 mg/kg diazepam to curtail seizures and reduce morbidity and mortality. For mice in Control group, all the injections were performed with same volume of saline at the same time points. Afterward, all the mice were raised under the same condition. Mice in Control and TLE groups were used for qPCR detection: six randomly selected mice in each group were sacrificed 4 and 12 h after model establishment, respectively and brain tissues were collected for qPCR validation of miR-155 expression as described following. Mice in CK and antagonist groups were housed for 7 days to determine the effect of antagonist on the survival of mice. Another 24 mice were grouped as described above (six for each group) for detection of association between miR-155 and BDNF. The detail experimental design was shown in Supplementary Figure S1.

### Quantitative Real-Time PCR

For qPCR detection, whole RNA in brain tissues from mice was extracted using RNA simple Total RNA Kit according to the manufacturers’ instruction (Calt. No.DP419, TIANGEN, Beijing, China). β-actin was selected as the internal reference gene. Then the whole RNA was reversely transcribed to cDNA templates using Super M-MLV reverse transcriptase (Calt. No. RP6502, BioTeke, Beijing, China). The final qPCR reaction mixture of volume 20 μL consisted of 10 μL of SYBR GREEN mastermix, 0.5 μL of each primers (*miR-155*, forward: 5′-CAGCCTACACGGTGGGAGC-3′, reverse: 5′-CTGCTCTGAGTCATTGTGCTGG-3′; *BDNF*, forward: 5′-GGTGGTGTAAGCCGCAAAG-3′, reverse: 5′-GTGTCAGCCAGTGATGTCG-3′; β*-actin* forward: 5′-CTTAGTTGCGTTACACCCTTTCTTG-3′, reverse: 5′-CTGTCACCTTCACCGTTCCAGTTT-3′), 1 μL of the cDNA template, and 8 μL of RNAse free H_2_O. Amplification parameters were as follows: denaturation at 95°C for 10 min, followed by 40 cycles at 95°C for 10 s, 60°C for 20 s, and 72°C for 30 s. Relative expression levels of the targeted molecules were calculated with Data Assist Software version 3.0 (Applied Biosystems/Life Technologies) according to the expression of 2^-ΔΔct^.

### Electroencephalogram (EEG) and Behavior Analyses

For recoding of EEG, experimental mice were anesthetized with pentobarbital sodium (50 mg/kg) and placed in stereotaxic frame. Two skull-mounted recording electrodes were subcutaneously placed in the bregma and occiput of the left ear of the mouse, and another skull-mounted recording electrode was placed on right ear of the mouse. All the electrodes were assembly fixed in place with dental cement during the surgical process. Animals were housed under the same condition and EEGs of mice were recorded and analyzed using BL-420F Biological Functional System (Techman Soft, Chengdu, China) 4 h and 3 days after model establishment according to the manufacturer’s instruction. The differences in spike and frequency between different groups were analyzed. Three days after model establishment, the severity of convulsions of mice in each group was analyzed using Racine classification (Racine, 1972) following procedures of previous study in a blind fashion (Jimenez-Mateos et al., 2015). Then mice were sacrificed and brain tissues were collected for histological and molecular analyses.

### Western Blotting

The whole protein product in different groups was extracted using the Total Protein Extraction Kit according to the manufacturer’s instructions (Catl. No. WLA019, Wanleibio, China). β-actin was used as the internal reference. Concentration of protein samples was determined using the BCA method. 20 μL of protein (40 μg) was subject to a 10% sodium dodecylsulfate polyacrylamide gel electrophoresis (SDS-PAGE). Targeted proteins were transferred onto polyvinylidene difluoride (PVDF) sheets and the membranes were washed with TTBS for 5 min before incubation in skim milk powder solution for 1 h. Primary antibodies against BDNF (1:200) or β-actin (1:1000) were incubated with membranes at 4°C overnight. After four washes using TTBS, secondary HRP IgG antibodies (1:5000) were added into the mixture and incubated with the membranes for 45 min at 37°C. After additional six washes using TTBS, the blots were developed using Beyo ECL Plus reagent and the results were detected in the Gel Imaging System. The relative expression levels of BDNF in different samples were calculated with Gel-Pro-Analyzer (Media Cybernetics, USA).

### Immunohistochemical Detection

For immunohistochemical (IHC) detection, brain sections from different groups were placed in 60°C for 2 h before incubation with dimethylbenzene for dewaxing. The slides were hydrated with alcohol. Slides from different groups were fixed using methanol solution with 3% H_2_O_2_ and blocked with 1% goat serum for 15 min at room temperature. Primary anti-BDNF antibody (1:50) was added and incubated at 4°C overnight. After three cycles of 0.01 M PBS wash, 5 min for each cycle, secondary antibody (1:200) was added to the slides and placed at 37°C for 30 min before another three cycles of PBS wash. Slides were then incubated with HRP at 37°C for 30 min before three cycles of 5-min PBS washing. Then DAB was added to the slides and reacted for 3–10 min until the reaction was stopped by ddH_2_O. Slides were re-stained using hematoxylin and dehydrated. Percentage of positively stained cells and the staining intensity of the different groups were determined by observation under a microscope at 400× magnification.

### Regulation of BDNF by miR-155 in Mice Neuron Cells

Mice neuron cells were collected from fresh cerebral cortices of the newborn C57BL/6 mice and cultured routinely. p-EGFP-miR-155 (expression Vector of miR-155), control p-EGFP plasmid, and mutant p-EGFP-miR-155 were obtained from Genechem Biotech (Shanghai, China). Moreover, a fragment of the 3′ UTR of BNDF cDNA was inserted into pGL3 vector (vector containing firefly luciferase under the control of SV40 promoter, Promega) to form pGL3-BDNF plasmid. Concentration of mice neuron cells was adjusted to 1 × 10^4^/mL and incubated on slides for 24 h before co-transfection with different combinations of vectors using Lipofectamine 2000 reagent (Invitrogen) according to the manufacturer’s protocol, and subsequent selection was conducted using 400-μg/mL puromycin (Amresco, Solon, OH, USA). Grouping of cells were as following: (A) pGL3+p-EGFP group; (B) pGL3+p-EGFP-miR-155 group; (C) pGL3+mutant p-EGFP-miR-155 group; (D) pGL3-BDNF+p-EGFP group; (E) pGL3-BDNF+p-EGFP-miR-155 group; (F) pGL3-BDNF+mutant p-EGFP-miR-155 group. Dual luciferase assay was conducted to measure the firefly luciferase activity using Luciferase Report Gene Assay Kit (E1910, Promega). Then the activities of BDNF in healthy neuron cells, cells transfected with p-EGFP-miR-155 (expression Vector of miR-155), and cells transfected with p-EGFP plasmid were determined with qPCR and Western blot as described above.

### Statistical Analysis

All the data were expressed in the form of mean ± SD. ANOVA and *Post doc* multiple comparisons using LSD method were conducted using a general liner model (GLM). Two-group data were compared using Student’s *t*-test. Difference between proportions of survival mice in Control and TLE groups was analyzed with a log-rank test. Significance was accepted at *P* < 0.05. All the statistical analysis and graph manipulation were conducted using GraphPad Prism 6 (GraphPad Software, San Diego, CA, USA).

## Results

### Induction of TLE Up-Regulated the Expression of miR-155 *In Vivo*

**FIGURE 1:**
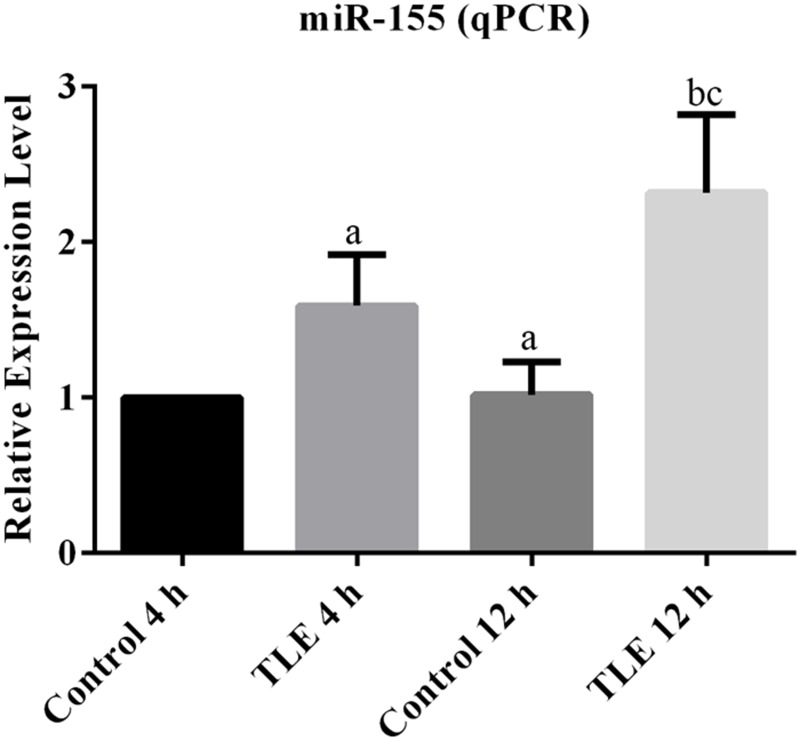
**Induction of TLE up-regulated the transcription of *miR-155* and the effect was time-dependent.** “a” significantly different from Control 4 h, *P* < 0.05. “b” significantly different from TLE 4 h, *P* < 0.05. “c” significantly different from Control 12 h, *P* < 0.05.

The expression of miR-155 was firstly investigated with mice from Control and TLE group at two time points. For brain tissues sampled 4 h after model establishment, there was significantly difference between the relative expression values of the two groups (*P* < 0.05; **Figure 1**). For brain samples from 12 h after TLE induction, the average miR-155 level in TLE group was higher than that in Control group, and the difference was statistically significant (*P* < 0.05; **Figure 1**). In addition, the inducing effect of TLE on miR-155 expression was time dependent in that the difference between samples from two time points in TLE group was statistically different (*P* < 0.05; **Figure 1**).

### Administration of miR-155 Antagonist Reduced Mice Mortality Caused by TLE

**FIGURE 2:**
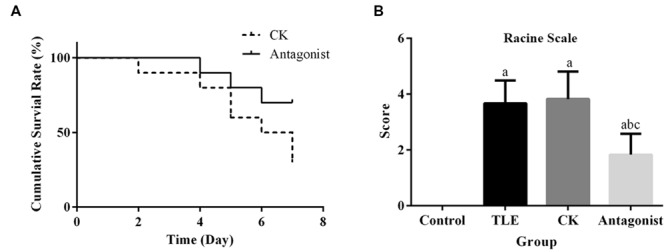
**Administration of specific miR-155 antagonist improved the survival rate and seizures in TLE mice. (A)** Comparison of survival curves between antagonist and CK group, treatment of miR-155 antagonist increased the survival rate of TLE mice; **(B)** quantitative analysis results of Racine scores, treatment of miR-155 antagonist decreased the seizures in TLE mice. “a” significantly different from Control group, *P* < 0.05. “b” significantly different from TLE group, *P* < 0.05. “c” significantly different from CK group, *P* < 0.05.

Survival curves of mice in CK and antagonist groups were shown in **Figure 2A**. In CK group, only three mice survived until the 7th day after TLE induction. Contrary to the result of CK group, seven mice in antagonist group survived to the ending point of the detection. Although, the survival difference between the two groups was not statistically different based on the analysis of log-rank test (*P* = 0.053), the results still indicated the potential of miR-155 antagonist to reduce TLE-induced mortality in mice.

### Administration of miR-155 Alleviated Epilepticus Symptoms Related to TLE

Seizure behavior after TLE induction was analyzed using a Racine scale. Three days after model establishment, TLE mice injected with miR-155 antagonist displayed significantly reduced seizure scores compared with those received saline or non-targeting antagonist injections (**Figure 2B**). No significant difference was detected for the average scores between mice in TLE and CK groups.

The results of EEG recordings were recorded and shown in Supplementary Figure S2 and Supplementary Table S1. No significant effect of miR-155 antagonist on the brain activity of TLE mice could be observed based on data derived from 4 h. For recordings 3 days after TLE induction, it was demonstrated that brain activities of miR-155 antagonist treated mice was similar to that of health mice Although EEG detection didn’t provide solid evidence in differentiating TLE from non-TLE cases, it might suggest some difference in the posterior background activities in brains of miR-155 antagonist treated mice as compared to those with TLE (Supplementary Figure S2 and Supplementary Table S1).

### Administration of miR-155 Antagonist Up-Regulated the Expression of BDNF Both at mRNA and Protein Levels

**FIGURE 3:**
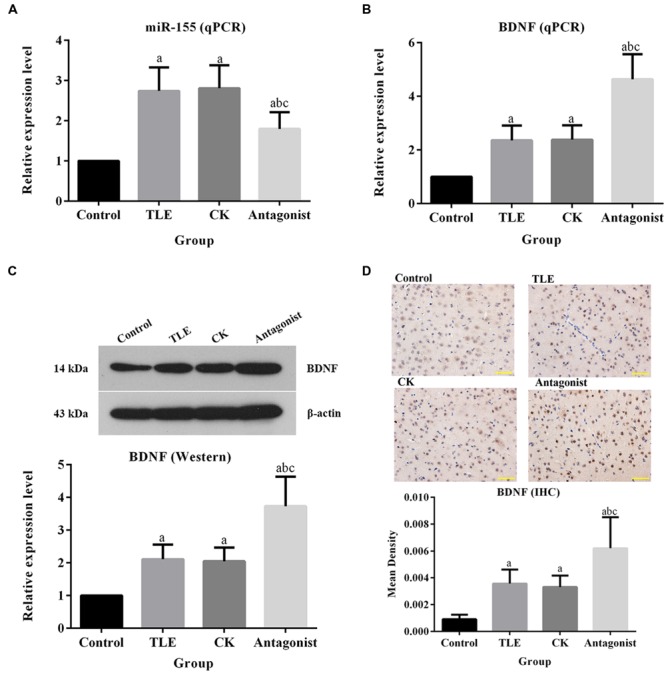
**Administration of miR-155 antagonist up-regulated the expression of BDNF. (A)** Quantitative analysis results of qPCR validation of miR-155. **(B)** Quantitative analysis results of qPCR validation of BDNF, treatment of miR-155 antagonist increased the production of BDNF at mRNA level. **(C)** Quantitative analysis results and representative images of Western blotting assay of BDNF, treatment of miR-155 antagonist increased the production of BDNF at protein level. **(D)** Quantitative analysis results and representative images of IHC assay of BDNF, the BDNF positive-stained cells were characterized by brownish-yellow particles and treatment of miR-155 antagonist increased the production of BDNF. “a” significantly different from Control group, *P* < 0.05. “b” significantly different from TLE group, *P* < 0.05. “c” significantly different from CK group, *P* < 0.05. Scale bar: 50 μm.

**FIGURE 4:**
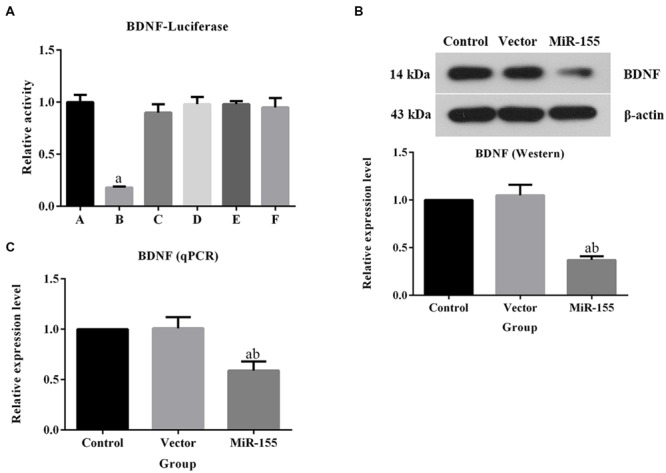
**Overexpression of miR-155 in neuron cells inhibited the expression of BDNF. (A)** Quantitative analysis results of dual luciferase assay. (A) pGL3+p-EGFP group; (B) pGL3+p-EGFP-miR-155 group; (C) pGL3+mutant p-EGFP-miR-155 group; (D) pGL3-BDNF+p-EGFP group; (E) pGL3-BDNF+p-EGFP-miR-155 group; (F) pGL3-BDNF+mutant p-EGFP-miR-155 group. **(B)** Quantitative analysis results of qPCR validation of BDNF, overexpression of miR-155 inhibited the production of BDNF at mRNA level. **(C)** Quantitative analysis results and representative images of Western blotting assay of BDNF, overexpression of miR-155 inhibited the production of BDNF at protein level. For dual luciferase assay, “a” represents significantly different from pGL3+p-EGFP group, *P* < 0.05. For qPCR and Western blotting assays, “a” represents significantly different from Control group and “b” represents significantly different from Vector group, *P* < 0.05.

As shown in **Figures 3A,B**, the pre-treatment of miR-155 antagonist led to increase in the transcription *BDNF*. The relative expression of *BDNF* in antagonist group was ca. twofold higher than those in TLE and CK groups, and the difference was statistically significant (*P* < 0.05). Similar pattern was also observed in Western blot (**Figure 3C**). For IHC detection, the BDNF positive-stained cells were characterized by brownish-yellow particles and the expression of the indicator were up-regulated in miR-155 antagonist treated group, which was identical to the results of qPCR and Western blotting (**Figure 3D**). To further explain the modulation of miR-155 on *BDNF*, a dual luciferase assay was conducted. Reporter assay revealed that overexpression of miR-155 significantly suppressed the activity of BDNF plasmid in neuron cells (*P* < 0.05; **Figure 4A**), indicating that miR-155 directly modulate BDNF expression by binding to 3′ UTR of the gene. The detection of BDNF production in neuron cells using qPCR and Western blotting also drawn similar conclusions as that of dual luciferase assay. At mRNA level, overexpression of miR-155 in cells inhibited the expression of BDNF (**Figure 4B**), and the difference between Control group and miR155 overexpressed group was statistically significant (*P* < 0.05). Additionally, at protein level, the redundant expression of miR155 resulted in a significant decrease of BDNF expression compared with that in Control group (*P* < 0.05; **Figure 4C**).

## Discussion

MiR-155 is a typical multifunction miR and plays crucial roles in numerous biological processes (Faraoni et al., 2009). Plenty of effect has been made to investigate the function of miR-155 in neoplastic, cardiovascular, and viral infection diseases (Esquela-Kerscher and Slack, 2006; Martin et al., 2007; Tili et al., 2007), results of which imply the great value of miR-155 for developments of novel treatment modalities against types of devastating diseases. Thus, in the present study, the dynamic expression of miR-155 in a lithium-pilocarpine-induced TLE mice model and the potential of miR-155 as a promising therapy against epilepsy were explored. It was found that inhibition of miR-155 with specific antagonist resulted in the alleviation of seizure behavior induced by TLE induction and reduced mortality in TLE mice. Moreover, our data also evidently showed that blocking or overexpression of miR-155 altered the production of BDNF, representing a close association between the two indicators in the onset of epilepsy.

Previous study of Jimenez-Mateos et al. (2015) reported that *in vivo* silencing of miR-134 resulted in a potent anticonvulsant effect through increasing volume of neuron spines in hippocampal CA3 pyramid, inferring the involvement of miRs in the pathophysiology of epilepsy. In the present study, the level of miR-155 was significantly up-regulated by TLE induction, which supported the conclusion that expression of miR-155 was abnormally high in patients of MTLE (Ashhab et al., 2013). Based on the EEG and behavior analyses, up-regulation of miR-155 in TLE mice was accompanied by the deterioration of the EEG performance and seizure features. Fortunately, in mice treated with miR-155 antagonist, the onset of seizures was delayed and the seizure power was reduced as well, representing an effectively anticonvulsant capability of miR-155 antagonist.

Although, results in the current study cannot explicitly explain the mechanism driving the anticonvulsant effect of miR-155 antagonist, it might highlight some aspects which had been long ignored. As shown by the detection of BDNF in TLE mice, a negative regulating effect of miR-155 on BDNF was validated. This result was contrary to some reports regarding the role BDNF in epilepsy (Heinrich et al., 2011; Wang et al., 2011). Previous studies demonstrated an increase of BDNF in epilepsy patients: the molecule could induce epileptogenesis by selectively activating pathways such as TrkB signaling (Heinrich et al., 2011). BDNF is a member of the neutrophin family of growth factors and involved in numerous aspects of development, degeneration, and differentiation of central nervous system (Tongiorgi et al., 2004). MiRs including miR-155 are capable of directly repressing BDNF through binding to their predicted sites in BDNF 3′ UTR (Varendi et al., 2014). In the current study, the abnormally high level of BDNF in epilepsy patients and the negative regulation of miR-155 on BDNF were verified. However, taken this information together, it seemed that the study had come to a dead end based on the existing theories. According to the results of EEG and behavior analyses, the up-regulation of BDNF by miR-155 antagonist was associated with the amelioration of seizures, representing a paradox considering the formerly reported expression pattern of BDNF in epilepsy (Heinrich et al., 2011; Wang et al., 2011). Nevertheless, up-regulation of BDNF might also be a contributor to the alleviation of epilepsy. Although, a correlation between seizure activity in TLE patient brains and BDNF production existed, this might represent a BDNF-dependent activation of neuropeptide Y (Takahashi et al., 1999). Level of neuropeptide Y increases in various brain regions after limbic seizures and has an anticonvulsant-activity in experimental animals (Bellmann et al., 1991; Erickson et al., 1996; Woldbye et al., 1997). Since, this increase in neuropeptide Y was preceded by increase of BDNF after seizures, miR-155 antagonists might exert their function by activating BDNF expression and subsequently inducing neuropeptide Y production.

Both *in vivo* and *in vitro* experiments were performed to assess the potential of miR-155 as an anticonvulsant target in the present study. Based on our findings, considerable improving effect of miR-155 antagonist on seizures was detected and it was hypothesized that miR-155 antagonist might firstly induce the expression of BDNF and then increase the synthesis of neuropeptide Y to protect brain tissues from the impairments of epileptogenesis. However, other mechanisms involved in this treatment might also exist. To further promote the application of specific miR antagonists in treatment of brain disorders, more comprehensive work needed to be conducted in the future.

## Author Contributions

HG and Song Li, Sheng Li planned experiments, performed experiments, and wrote the draft; FS, ZZ, GQ, TL, and JQ performed experiments and analyzed data; JW and HS contributed reagents; ZC planned experiments, revised the draft, and approved the final submission.

## Conflict of Interest Statement

The authors declare that the research was conducted in the absence of any commercial or financial relationships that could be construed as a potential conflict of interest.
