# Supplementary material for: Antagonist Targeting microRNA-155 Protects against Lithium-Pilocarpine-Induced Status Epilepticus in C57BL/6 Mice by Activating Brain-Derived Neurotrophic Factor
Source: Front Pharmacol. 2016 May 31;7:129. doi: 10.3389/fphar.2016.00129 (PMC4885878; doi:10.3389/fphar.2016.00129)
Supplement: Supplementary file 1 [file Data_Sheet_1.DOC]

**Figure S1** Illustrated experiment design of in vivo assay. A, animal grouping and examination timeline of RT-qPCR validation of miR155 and survival analysis. B, animal grouping and examination timeline of EEG recording, IHC, and western blotting (WB).

**Figure S2** Representative images of EEG recordings.

**Table S1 Detail data of EEG recording**

| Parameter | 4 hour | | | | 3 day | | | |
| --- | --- | --- | --- | --- | --- | --- | --- | --- |
|  | Control | TLE | CK | Antagonist | Control | TLE | CK | Antagonist |
| Frequency (Hz) | 9.0 | 3 | 8 | 4 | 2 | 9 | 9 | 2 |
| Highest Amplitude (μV) | 95.9 | 200.0 | 145.8 | 200 | 73.9 | 98.3 | 66.2 | 53.0 |
| Lowest Amplitude (μV) | -120.6 | -86.6 | -122.8 | 15.6 | -132.9 | -133.9 | -108.5 | -161.0 |
| Average Amplitude (μV) | -17.58 | 77.9 | 1.42 | 113.8 | -25.7 | 5.0 | -14.3 | -22.3 |
