# Supplementary figures and images for: Antagonist Targeting microRNA-155 Protects against Lithium-Pilocarpine-Induced Status Epilepticus in C57BL/6 Mice by Activating Brain-Derived Neurotrophic Factor
Source: Front Pharmacol. 2016 May 31;7:129. doi: 10.3389/fphar.2016.00129 (PMC4885878; doi:10.3389/fphar.2016.00129)

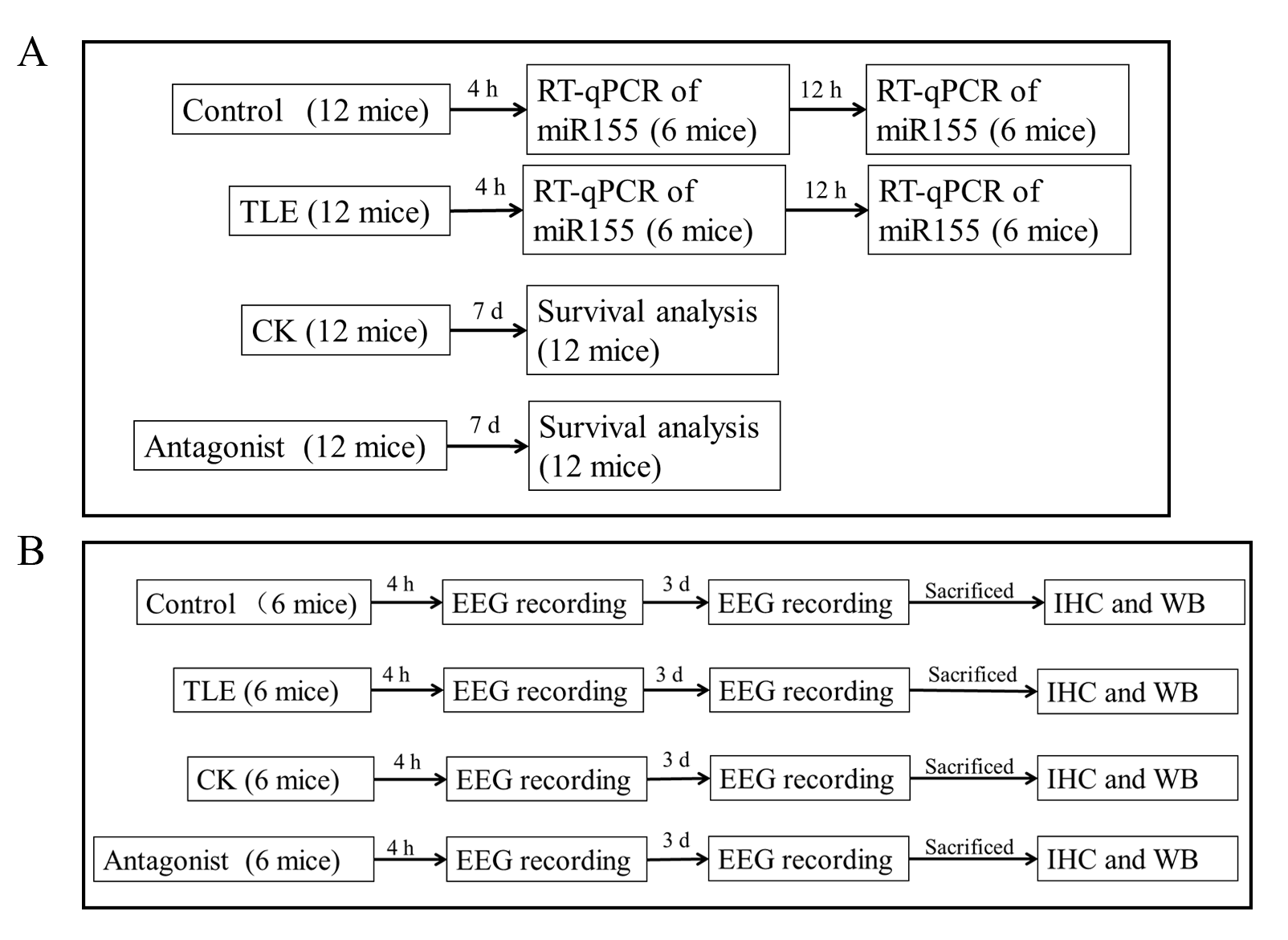

Supplement: Supplementary file 2 [file Image_1.TIF]

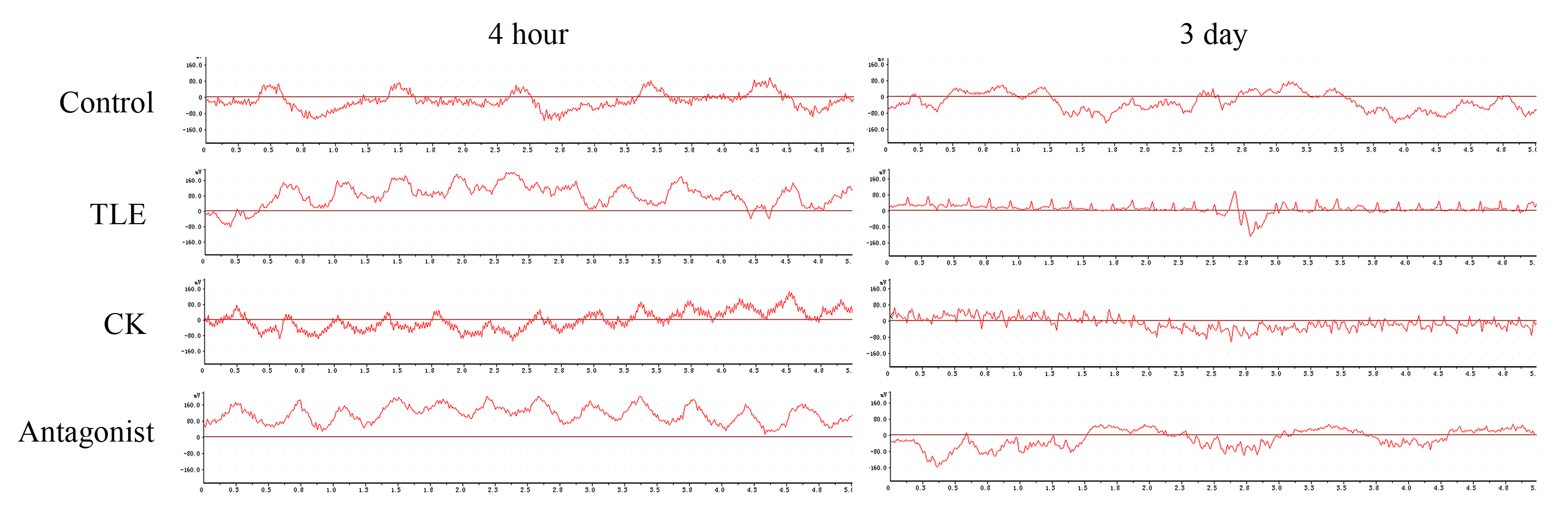

Supplement: Supplementary file 3 [file Image_2.TIF]
